# *Cis versus trans* arrangement of di­thio­carbazate ligands in bis-chelated Ni and Cu complexes

**DOI:** 10.1107/S205698902000506X

**Published:** 2020-04-21

**Authors:** Khurshida Begum, Sabina Begum, Chanmiya Sheikh, Ryuta Miyatake, Ennio Zangrando

**Affiliations:** a Department of Physics, Shahjalal University of Science and Technology, Sylhet 3114, Bangladesh; b Department of Chemistry, Shahjalal University of Science and Technology, Sylhet 3114, Bangladesh; c Department of Applied Chemistry, Faculty of Engineering, University of Toyama, Gofuku, Toyama 3190, Japan; d Center for Environmental Conservation and Research Safety, University of Toyama, Gofuku, Toyama 3190, Japan; e Department of Chemical and Pharmaceutical Science, via Giorgieri 1/34127, Trieste, Italy

**Keywords:** crystal structure, di­thio­carbazate ligand, nickel(II) complex, copper(II) complex, *cis*-*trans* configuration

## Abstract

The structures are described of two bis-chelated metal complexes of nickel(II) and copper(II) with *S*-*n*-hexyl 3-(1-phenyl­ethyl­idene)di­thio­carbazate Schiff bases in a *cis* configuration, namely, bis­[*S*-*n*-hexyl 3-(1-phenyl­ethyl­idene)di­thio­carbazato-κ^2^ *N* ^3^,*S*]nickel(II), [Ni(C_15_H_21_N_2_S_2_)_2_], and bis­[*S*-*n*-hexyl 3-(1-phenyl­ethyl­idene)di­thio­carbazato-κ^2^ *N* ^3^,*S*]copper(II), [Cu(C_15_H_21_N_2_S_2_)_2_]. In both complexes, the metals have distorted square-planar geometries. A search in the Cambridge Structural Database [Groom *et al.* (2016 ▸). *Acta Cryst.* B**72**, 171–179] for bis-chelated nickel(II) and copper(II) complexes with similar Schiff bases retrieved 55 and 36 hits for the two metals, respectively. An analysis of the geometrical parameters of complexes showing *cis* and *trans* configurations is reported and the values compared with those for the complexes described in this work.

## Abstract 2

Two bis-chelated metal complexes of nickel(II) and copper(II) with *N*,*S* Schiff bases in a *cis* configuration are presented and compared with similar species in the CSD having *trans-*configured ligands.

## Chemical context   

Thio­semicarbazones, semicarbazones, hydrazide/hydrazones and di­thio­carbazate Schiff bases and their complexes have been widely studied for their significant bioactivities and pharmacological properties (Beraldo *et al.* 2004 ▸; Altıntop *et al.*, 2016 ▸). The presence of hard nitro­gen and soft sulfur atoms enable these ligands to react with both transition and main-group metals (Arion, 2019 ▸) and transition-metal complexes derived from these N,S Schiff bases occupy a central role in the area of coordination chemistry. The nature of the long alkyl substituent chains, when present, may play a role in determining the liquid crystalline behavior of the complexes and thus their mesomorphic potential (Tomma *et al.*, 2018 ▸; Lai *et al.*, 1998 ▸).
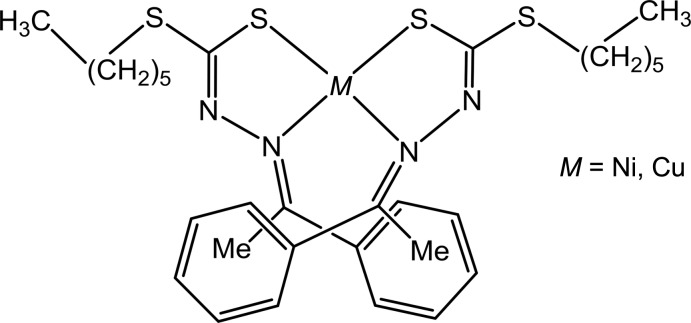


Therefore, considering the above facts and in a continuation of our inter­est in this field (Zangrando *et al.*, 2017 ▸), the present work reports a study on the synthesis and structural characterization of Ni^II^ and Cu^II^ complexes **1** and **2** with the Schiff base derived from *S*-*n*-hexyl­dithio­carbazate and aceto­phenone (H*L*). The single crystal X-ray structures of these distorted square-planar complexes of nickel and copper, Ni*L*_2_ and Cu*L*_2_, show *cis* configurations of the ligands. Since similar complexes can show both *cis* and *trans* configurations, we report herein a comparison with the geometry of structurally characterized complexes retrieved from the Cambridge Structural Database (Groom *et al.*, 2016 ▸).

## Structural commentary   

### Structure of complex 1   

**Figure 1:**
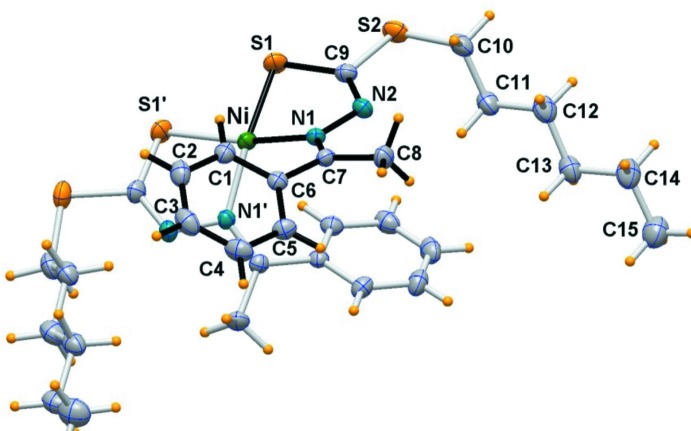
*ORTEP* view (50% probability ellipsoids) of the nickel(II) complex (**1**) with the labeling scheme for the asymmetric unit. (Primed atoms are related by the symmetry operation −*x* + 1, *y*, −*z* + 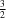).

In the Ni*L*_2_ complex, the nickel atom is located on a crystallographic twofold axis and exhibits a distorted square-planar geometry. An *ORTEP* drawing of the complex is depicted in Fig. 1 ▸ and selected geometrical data are reported in Table 1 ▸. The two Schiff bases, in their deprotonated imino thiol­ate form, are coordinated through the β-nitro­gen atom, N1, and the thiol­ate sulfur atom, S1, donors to the metal center in a *cis*-planar configuration. The Ni—S and Ni—N bond distances are 2.1600 (4) and 1.9295 (10) Å, respectively, with an S—Ni—N chelating angle of 85.68 (3)°.

The square-planar geometry is tetra­hedrally distorted and the dihedral angle formed by the mean planes through the two five-membered rings is 19.46 (5)°. The distortion from a planar arrangement is effected in order to circumvent steric clashes between the phenyl rings due to the *cis* configuration of the ligands.

### Structure of complex 2   

**Figure 2:**
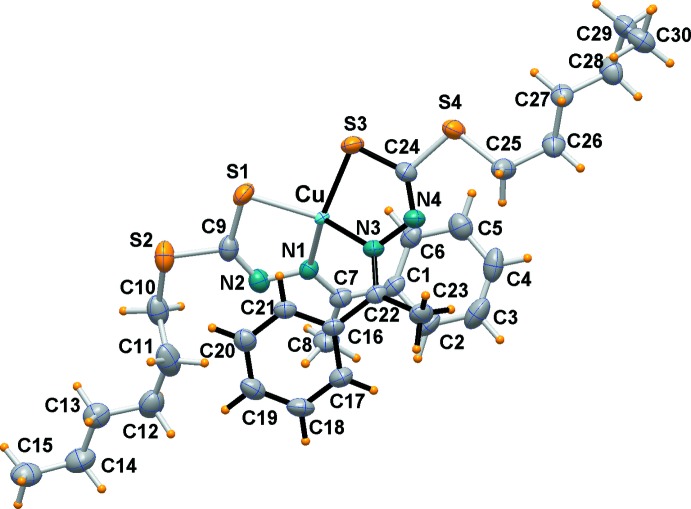
*ORTEP* view (50% probability ellipsoids) of the copper(II) complex (**2**).

In Cu*L*_2_, the whole copper(II) complex is crystallographically independent although it exhibits pseudo twofold symmetry. An *ORTEP* view is shown in Fig. 2 ▸, and selected geometrical data are reported in Table 2 ▸. The arrangement of the ligands is similar to that of the nickel derivative, but a different conformation of the two alkyl chains leads to a lack of symmetry. Here the Cu—S and Cu—N bond distances are 2.2299 (9) and 2.2414 (9) Å, and 2.023 (3) and 2.020 (3) Å, respectively, while the chelating angles are similar at 85.43 (8) and 85.37 (8)°. The square-planar geometry shows a more significant tetra­hedral distortion than is found in complex **1**, having a dihedral angle between the two five-membered rings of 40.41 (12)°. It is worth noting that compared to similar ligands in their uncoordinated state (see for example Begum *et al.*, 2015 ▸), a rotation about the C9—N2 by 180° is observed in the metal complexes in order to allow the N,S chelating behavior towards the metal.

The configuration assumed by the ligands in each complex leads the phenyl hydrogen atoms to sit above and below the metal centres with a separation of ∼2.6 Å, indicating the presence of *M*⋯H intra­molecular inter­actions.

## Supra­molecular features   

**Figure 3:**
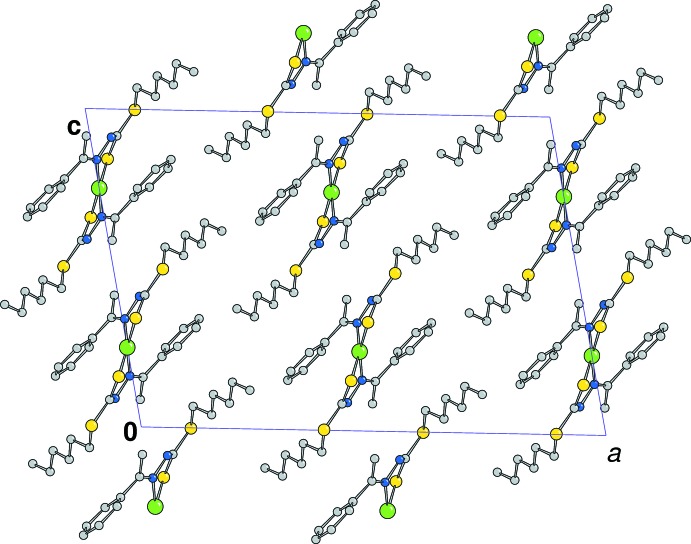
The crystal packing of the Ni complex viewed down the *b* axis (H atoms are not shown for clarity).

Figs. 3 ▸ and 4 ▸ display the crystal packing of the two complexes. The slightly shorter distance between the nickel ions in **1** (8.337 Å) compared to that of the copper atoms in **2** (8.518 Å) is likely the result of the different conformations of the alkyl chains. In both structures no significant π–π inter­actions involving phenyl rings are detected. C—H⋯π inter­actions are observed in **1** (Table 3 ▸) but no such inter­actions are observed in **2**.

## Database survey   

Table 3 ▸ reports the mean values of the coordination bond lengths and angles of nickel(II) and copper(II) complexes bis-chelated by di­thio­carbazate ligands, as retrieved from the CSD (version 5.40, update of August 2019; Groom *et al.*, 2016 ▸). Whereas the number of *trans*-configured nickel complexes is higher than the number of *cis* complexes, for copper, the numbers of *trans*- and *cis*-planar complexes are almost equal. The Ni—N, Cu—N and Cu—S bond distances are comparable in the *cis* and *trans* isomers, while for the Ni–S bond distances, a slight shorter distance is observed for the *cis* isomers than for the *trans* isomers [2.157 (8) *vs* 2.174 (8) Å]. More significant is the dihedral angle between the five-membered rings of the chelating ligands, which has a value close to 0° in both the *trans*-configured Ni and Cu complexes, while in the *cis*-Ni complexes the angle does not exceed 31°, and in the *cis*-Cu complexes, the smallest value observed is 32.27°, indicating a propensity for copper(II) to assume a tetra­hedral configuration. In fact, in some of the *cis* copper complexes in Table 4 ▸, the metal is present in effectively a tetra­hedral geometry with a dihedral angle between the five-membered rings of *ca* 80° (Mondal *et al.*, 2014 ▸; Santra *et al.*, 2016 ▸; Tarafder *et al.*, 2008 ▸). Another feature is a slight difference between the N—Ni—N and S—Ni—S angles in the *cis* complexes (100.39 and 92.30°, respectively), while the N—Cu—N and S—Cu—S angles are comparable (*ca* 106°) in the *cis*-Cu complexes.

Overall, it is difficult to assess what drives particular complexes to assume either a *cis* or a *trans* configuration upon crystallization and the most plausible reason may arise from crystal-packing requirements. Similar derivatives having thienyl­methyl­ene instead of the phenyl­ethyl­idene fragments crystallize with a *trans* configuration (Begum *et al.*, 2016 ▸).

## Synthesis of the Schiff base ligand   

Hydrazine hydrate (2.50 g, 0.05 mol, 99%) was added to an ethano­lic solution (30 ml) of KOH (2.81 g, 0.05 mol) and the mixture was stirred at 273 K for 45 min. To this solution, carbon di­sulfide (3.81 g, 0.05 mol) was added dropwise under constant stirring for one h. Then 1-bromo­hexane (8.25 g, 0.05 mol) was added dropwise at 273 K under vigorous stirring for another hour. Finally, aceto­phenone (6.00 g, 0.05 mol) in ethanol (2.0 ml) was added and the mixture refluxed for 30 minutes. The hot mixture was filtered and then the filtrate cooled to 273 K to give a precipitate of the Schiff base product, which was recrystallized from ethanol at room temperature and dried in a vacuum desiccator over anhydrous CaCl_2_.

### Synthesis of the Ni complex, 1   

A solution of nickel(II) acetate tetra­hydrate (0.06 g, 0.25 mmol, 7 mL methanol) was added to a solution of the ligand, (0.147 g, 0.5 mmol, 10 mL methanol). The resulting mixture was stirred at room temperature for five h. An olive green precipitate was formed, filtered off, washed with methanol and dried in vacuo over anhydrous CaCl_2_. Dark reddish brown single crystals of the compound, suitable for X-ray diffraction, were obtained by slow evaporation from a mixture of chloro­form and toluene (5:1). Yield 85%. ESI-MS (FAB) calcd. *m/z* for C_30_H_42_N_4_S_4_Ni + H^+^: 644.1646 amu, found 645.1724 amu. M.p. 374 K.

### Synthesis of the Cu complex, 2   

The copper complex was prepared by a similar method to that used for nickel in the presence of Cu(CH_3_COO)_2_·H_2_O. Dark reddish brown single crystals of the compound, suitable for X-ray diffraction, were obtained by slow evaporation from a mixture of chloro­form and aceto­nitrile (4:1). Yield 83%. ESI-MS (FAB) calcd. *m/z* for C_30_H_42_N_4_S_4_Cu + H^+^: 649.1588 amu, found 650.1665 amu. M.p. 418 K.

## Refinement details   

Crystal data, data collection and structure refinement details are summarized in Table 5 ▸. The hydrogen atoms were included as riding contributions with fixed isotropic displacement parameters in idealized positions [C—H = 0.95–0.99 Å; *U*_iso_(H) = 1.2 or 1.5*U*_eq_(C)]. The structure of **2** was refined as an inversion twin.

## Supplementary Material

Crystal structure: contains datablock(s) I, II, global. DOI: 10.1107/S205698902000506X/cq2035sup1.cif

Structure factors: contains datablock(s) I. DOI: 10.1107/S205698902000506X/cq2035Isup2.hkl

Structure factors: contains datablock(s) II. DOI: 10.1107/S205698902000506X/cq2035IIsup3.hkl

CCDC references: 1057808, 1403802

Additional supporting information:  crystallographic information; 3D view; checkCIF report

## Figures and Tables

**Figure 4:**
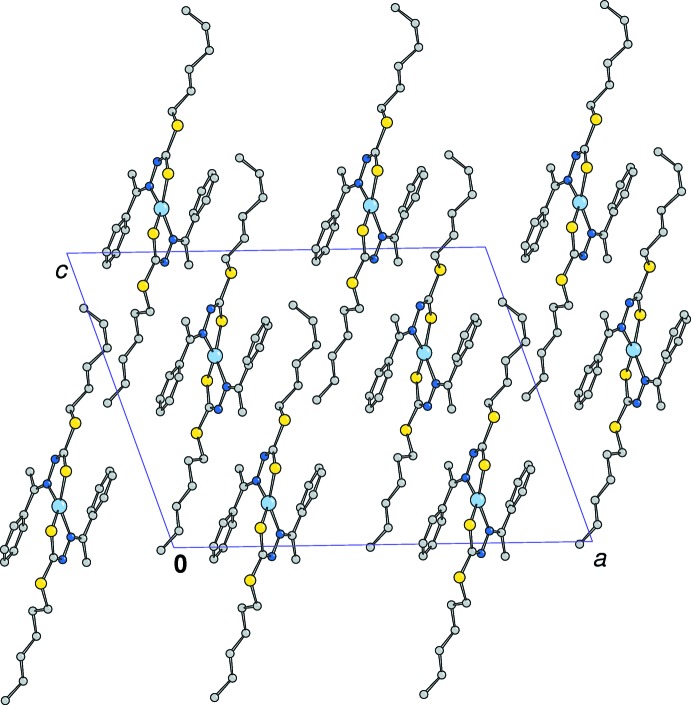
The crystal packing of the Cu complex viewed down the *b* axis (H atoms are not shown for clarity).

**Table 1:** Selected geometric parameters (Å, °) for **1** [Chem scheme1]

Ni1—N1	1.9295 (10)	Ni1—S1	2.1600 (4)
			
S1—Ni1—S1^i^	93.12 (2)	N1—Ni1—S1^i^	163.99 (3)
N1—Ni1—S1	85.68 (3)	N1—Ni1—N1^i^	99.79 (6)

Symmetry code: (i) 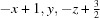.

**Table 2:** Selected geometric parameters (Å, °) for **2** [Chem scheme1]

Cu1—N1	2.023 (3)	Cu1—S1	2.2299 (9)
Cu1—N3	2.020 (3)	Cu1—S3	2.2414 (9)
			
S1—Cu1—S3	98.53 (4)	N1—Cu1—S3	152.51 (8)
N1—Cu1—S1	85.43 (8)	N3—Cu1—S3	85.37 (8)
N3—Cu1—S1	149.66 (8)	N1—Cu1—N3	104.90 (11)

**Table 3:** C—H⋯π interation (Å, °) in **1** [Chem scheme1] *Cg* is the centroid of the C1–C6 ring.

*D*—H⋯*A*	*D*—H	H⋯*A*	*D*⋯*A*	*D*—H⋯*A*
C14—H14*A*⋯*Cg* ^ii^	0.99	2.75	3.5892 (18)	143

Symmetry code: (ii) 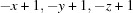.

**Table 4:** Coordination bond lengths and angles (Å, °) in the di­thio­carbazate nickel and copper complexes with *trans* and *cis* configurations retrieved from the CSD α is the dihedral angle between the five-membered rings of the chelating ligands.

	*trans*-Ni*L*_2_	*cis*-Ni*L*_2_	*trans*-Cu*L*_2_	*cis*-Cu*L*_2_
No. of structures	32	23	19	17
*M*—N mean	1.920 (13)	1.924 (20)	1.996 (37)	2.013 (22)
*M*—N range	1.878–1.952	1.851–1.995	1.923–2.043	1.986–2.066
*M*—S mean	2.174 (8)	2.157 (8)	2.244 (37)	2.240 (17)
*M*—S range	2.145–2.195	2.141–2.177	2.166–2.281	2.215–2.287
N—*M*—N mean	179.21	100.39	179.34	105.76
S—*M*—S mean	178.39	92.30	179.01	106.28
α mean	1.75	21.25	0.80	50.25
α range	0.00–19.41	10.24–30.10	0.00–10.93	32.27–81.61

**Table 5:** Experimental details

	**1**	**2**
Crystal data		
Chemical formula	[Ni(C_15_H_21_N_2_S_2_)_2_]	[Cu(C_15_H_21_N_2_S_2_)_2_]
*M*_r_	645.62	650.45
Crystal system, space group	Monoclinic, *C*2/*c*	Monoclinic, *C* *c*
Temperature (K)	173	173
*a*, *b*, *c* (Å)	23.9721 (5), 8.3967 (2), 16.6739 (3)	22.7441 (7), 8.8636 (3), 17.0117 (6)
β (°)	101.046 (1)	109.158 (1)
*V* (Å^3^)	3294.05 (12)	3239.53 (19)
*Z*	4	4
Radiation type	Mo *K*α	Mo *K*α
μ (mm^−1^)	0.87	0.96
Crystal size (mm)	0.38 × 0.30 × 0.07	0.23 × 0.10 × 0.03

Data collection		
Diffractometer	Rigaku R-AXIS RAPID	Rigaku R-AXIS RAPID
Absorption correction	Multi-scan (*ABSCOR*; Rigaku, 1995 ▸)	Multi-scan (*ABSCOR*; Rigaku, 1995 ▸)
*T*_min_, *T*_max_	0.684, 0.941	0.772, 0.976
No. of measured, independent and observed [*I* > 2σ(*I*)] reflections	15965, 3768, 3589	7274, 7274, 6505
*R*_int_	0.025	0.025
(sin θ/λ)_max_ (Å^−1^)	0.649	0.649

Refinement		
*R*[*F* ^2^ > 2σ(*F* ^2^)], *wR*(*F* ^2^), *S*	0.027, 0.081, 1.15	0.031, 0.074, 1.04
No. of reflections	3768	7274
No. of parameters	179	357
No. of restraints	0	2
H-atom treatment	H-atom parameters constrained	H-atom parameters constrained
Δρ_max_, Δρ_min_ (e Å^−3^)	0.32, −0.33	0.70, −0.22
Absolute structure	–	Refined as an inversion twin.
Absolute structure parameter	–	0.482 (10)

Computer programs: *RAPID-AUTO* and *CrystalStructure* (Rigaku, 2010 ▸), *SIR92* (Altomare *et al.*, 1994 ▸) and *SHELXL2014* (Sheldrick, 2015 ▸).

## supplementary crystallographic information

### Bis[S-n-hexyl 3-(1-phenylethylidene)dithiocarbazato-κ2N3,S]nickel(II) (I) Crystal data

**Table:**

[Ni(C_15_H_21_N_2_S_2_)_2_]	*F*(000) = 1368
*M**_r_* = 645.62	*D*_x_ = 1.302 Mg m^−^^3^
Monoclinic, *C*2/*c*	Mo *K*α radiation, λ = 0.71075 Å
*a* = 23.9721 (5) Å	Cell parameters from 4789 reflections
*b* = 8.3967 (2) Å	θ = 3.3–27.5°
*c* = 16.6739 (3) Å	µ = 0.87 mm^−^^1^
β = 101.046 (1)°	*T* = 173 K
*V* = 3294.05 (12) Å^3^	Prism, purple
*Z* = 4	0.38 × 0.30 × 0.07 mm

### Bis[S-n-hexyl 3-(1-phenylethylidene)dithiocarbazato-κ2N3,S]nickel(II) (I) Data collection

**Table:**

Rigaku R-AXIS RAPID diffractometer	3589 reflections with *I* > 2σ(*I*)
Detector resolution: 10.000 pixels mm^-1^	*R*_int_ = 0.025
ω scans	θ_max_ = 27.5°, θ_min_ = 3.3°
Absorption correction: multi-scan (ABSCOR; Rigaku, 1995)	*h* = −30→30
*T*_min_ = 0.684, *T*_max_ = 0.941	*k* = −10→10
15965 measured reflections	*l* = −21→21
3768 independent reflections	

### Bis[S-n-hexyl 3-(1-phenylethylidene)dithiocarbazato-κ2N3,S]nickel(II) (I) Refinement

**Table:**

Refinement on *F*^2^	Primary atom site location: structure-invariant direct methods
Least-squares matrix: full	Secondary atom site location: difference Fourier map
*R*[*F*^2^ > 2σ(*F*^2^)] = 0.027	Hydrogen site location: inferred from neighbouring sites
*wR*(*F*^2^) = 0.081	H-atom parameters constrained
*S* = 1.15	*w* = 1/[σ^2^(*F*_o_^2^) + (0.0458*P*)^2^ + 1.5849*P*] where *P* = (*F*_o_^2^ + 2*F*_c_^2^)/3
3768 reflections	(Δ/σ)_max_ = 0.002
179 parameters	Δρ_max_ = 0.32 e Å^−^^3^
0 restraints	Δρ_min_ = −0.33 e Å^−^^3^

### Bis[S-n-hexyl 3-(1-phenylethylidene)dithiocarbazato-κ2N3,S]nickel(II) (I) Special details

**Table:**

Geometry. All esds (except the esd in the dihedral angle between two l.s. planes) are estimated using the full covariance matrix. The cell esds are taken into account individually in the estimation of esds in distances, angles and torsion angles; correlations between esds in cell parameters are only used when they are defined by crystal symmetry. An approximate (isotropic) treatment of cell esds is used for estimating esds involving l.s. planes.

### Bis[S-n-hexyl 3-(1-phenylethylidene)dithiocarbazato-κ2N3,S]nickel(II) (I) Fractional atomic coordinates and isotropic or equivalent isotropic displacement parameters (Å^2^)

**Table:**

	*x*	*y*	*z*	*U*_iso_*/*U*_eq_	
Ni1	0.5000	1.00787 (2)	0.7500	0.02259 (8)	
S1	0.47077 (2)	1.18475 (4)	0.65741 (2)	0.03381 (10)	
S2	0.39340 (2)	1.11935 (4)	0.50136 (2)	0.03696 (11)	
N1	0.49459 (4)	0.85983 (12)	0.66033 (6)	0.0216 (2)	
N2	0.45428 (5)	0.89369 (13)	0.58858 (6)	0.0260 (2)	
C1	0.60886 (5)	0.81446 (16)	0.75593 (8)	0.0270 (3)	
H1	0.6020	0.9207	0.7371	0.032*	
C2	0.65594 (6)	0.78033 (19)	0.81594 (8)	0.0344 (3)	
H2	0.6814	0.8631	0.8376	0.041*	
C3	0.66588 (6)	0.6262 (2)	0.84440 (9)	0.0379 (3)	
H3	0.6981	0.6034	0.8857	0.046*	
C4	0.62895 (7)	0.50520 (18)	0.81276 (10)	0.0363 (3)	
H4	0.6354	0.3998	0.8331	0.044*	
C5	0.58238 (6)	0.53742 (16)	0.75130 (8)	0.0283 (3)	
H5	0.5579	0.4534	0.7284	0.034*	
C6	0.57150 (5)	0.69294 (15)	0.72306 (7)	0.0230 (2)	
C7	0.52256 (5)	0.72868 (14)	0.65686 (7)	0.0220 (2)	
C8	0.50807 (6)	0.61449 (16)	0.58659 (8)	0.0304 (3)	
H8A	0.4700	0.5709	0.5851	0.046*	
H8B	0.5358	0.5275	0.5935	0.046*	
H8C	0.5090	0.6706	0.5353	0.046*	
C9	0.44213 (6)	1.04365 (16)	0.58425 (8)	0.0269 (3)	
C10	0.38023 (7)	0.9506 (2)	0.43216 (8)	0.0369 (3)	
H10A	0.3617	0.9900	0.3776	0.044*	
H10B	0.4173	0.9042	0.4267	0.044*	
C11	0.34357 (6)	0.81914 (19)	0.45734 (8)	0.0347 (3)	
H11A	0.3070	0.8649	0.4654	0.042*	
H11B	0.3630	0.7733	0.5101	0.042*	
C12	0.33199 (7)	0.6872 (2)	0.39342 (9)	0.0406 (3)	
H12A	0.3093	0.7315	0.3425	0.049*	
H12B	0.3687	0.6502	0.3811	0.049*	
C13	0.30051 (6)	0.5448 (2)	0.41990 (9)	0.0359 (3)	
H13A	0.3242	0.4961	0.4689	0.043*	
H13B	0.2649	0.5824	0.4354	0.043*	
C14	0.28614 (7)	0.4183 (2)	0.35383 (10)	0.0437 (4)	
H14A	0.3219	0.3761	0.3408	0.052*	
H14B	0.2645	0.4686	0.3037	0.052*	
C15	0.25158 (8)	0.2802 (2)	0.37790 (12)	0.0532 (4)	
H15A	0.2721	0.2320	0.4286	0.080*	
H15B	0.2458	0.2002	0.3343	0.080*	
H15C	0.2146	0.3195	0.3863	0.080*	

### Bis[S-n-hexyl 3-(1-phenylethylidene)dithiocarbazato-κ2N3,S]nickel(II) (I) Atomic displacement parameters (Å^2^)

**Table:**

	*U*^11^	*U*^22^	*U*^33^	*U*^12^	*U*^13^	*U*^23^
Ni1	0.02882 (14)	0.01537 (12)	0.02345 (13)	0.000	0.00467 (9)	0.000
S1	0.0469 (2)	0.01795 (16)	0.03453 (18)	0.00174 (13)	0.00255 (15)	0.00438 (12)
S2	0.0389 (2)	0.03350 (19)	0.03533 (19)	0.00545 (14)	−0.00088 (15)	0.01283 (14)
N1	0.0239 (5)	0.0193 (5)	0.0213 (5)	−0.0007 (4)	0.0033 (4)	0.0022 (4)
N2	0.0268 (5)	0.0271 (5)	0.0226 (5)	0.0012 (4)	0.0012 (4)	0.0033 (4)
C1	0.0269 (6)	0.0278 (6)	0.0272 (6)	−0.0019 (5)	0.0077 (5)	−0.0024 (5)
C2	0.0265 (6)	0.0456 (8)	0.0306 (6)	−0.0044 (6)	0.0043 (5)	−0.0074 (6)
C3	0.0269 (7)	0.0561 (9)	0.0294 (6)	0.0087 (6)	0.0021 (5)	0.0032 (6)
C4	0.0313 (7)	0.0396 (8)	0.0386 (8)	0.0105 (5)	0.0078 (6)	0.0116 (6)
C5	0.0268 (6)	0.0244 (6)	0.0344 (7)	0.0033 (5)	0.0077 (5)	0.0024 (5)
C6	0.0222 (6)	0.0250 (6)	0.0231 (5)	0.0017 (4)	0.0071 (4)	−0.0004 (5)
C7	0.0243 (6)	0.0191 (5)	0.0231 (5)	−0.0020 (4)	0.0057 (4)	0.0005 (4)
C8	0.0367 (7)	0.0246 (6)	0.0291 (6)	0.0000 (5)	0.0039 (5)	−0.0056 (5)
C9	0.0282 (6)	0.0249 (6)	0.0274 (6)	0.0010 (5)	0.0052 (5)	0.0061 (5)
C10	0.0378 (8)	0.0472 (8)	0.0250 (6)	0.0008 (7)	0.0040 (5)	0.0060 (6)
C11	0.0303 (7)	0.0472 (8)	0.0263 (6)	−0.0003 (6)	0.0048 (5)	0.0002 (6)
C12	0.0391 (8)	0.0529 (9)	0.0310 (7)	−0.0024 (7)	0.0100 (6)	−0.0052 (7)
C13	0.0292 (7)	0.0482 (8)	0.0296 (7)	0.0020 (6)	0.0039 (5)	−0.0044 (6)
C14	0.0380 (8)	0.0549 (10)	0.0398 (8)	−0.0032 (7)	0.0116 (6)	−0.0123 (7)
C15	0.0461 (10)	0.0583 (11)	0.0551 (10)	−0.0091 (8)	0.0095 (8)	−0.0104 (9)

### Bis[S-n-hexyl 3-(1-phenylethylidene)dithiocarbazato-κ2N3,S]nickel(II) (I) Geometric parameters (Å, º)

**Table:**

Ni1—N1^i^	1.9295 (10)	C7—C8	1.5023 (17)
Ni1—N1	1.9295 (10)	C8—H8A	0.9800
Ni1—S1^i^	2.1600 (4)	C8—H8B	0.9800
Ni1—S1	2.1600 (4)	C8—H8C	0.9800
S1—C9	1.7443 (14)	C10—C11	1.519 (2)
S2—C9	1.7493 (13)	C10—H10A	0.9900
S2—C10	1.8163 (17)	C10—H10B	0.9900
N1—C7	1.2963 (16)	C11—C12	1.526 (2)
N1—N2	1.4151 (14)	C11—H11A	0.9900
N2—C9	1.2913 (17)	C11—H11B	0.9900
C1—C2	1.3872 (19)	C12—C13	1.524 (2)
C1—C6	1.3984 (17)	C12—H12A	0.9900
C1—H1	0.9500	C12—H12B	0.9900
C2—C3	1.383 (2)	C13—C14	1.521 (2)
C2—H2	0.9500	C13—H13A	0.9900
C3—C4	1.384 (2)	C13—H13B	0.9900
C3—H3	0.9500	C14—C15	1.523 (3)
C4—C5	1.390 (2)	C14—H14A	0.9900
C4—H4	0.9500	C14—H14B	0.9900
C5—C6	1.3956 (18)	C15—H15A	0.9800
C5—H5	0.9500	C15—H15B	0.9800
C6—C7	1.4794 (17)	C15—H15C	0.9800
			
S1—Ni1—S1^i^	93.12 (2)	N2—C9—S1	124.67 (10)
N1—Ni1—S1	85.68 (3)	N2—C9—S2	120.52 (11)
N1—Ni1—S1^i^	163.99 (3)	S1—C9—S2	114.81 (8)
N1—Ni1—N1^i^	99.79 (6)	C11—C10—S2	115.51 (10)
N1^i^—Ni1—S1^i^	85.68 (3)	C11—C10—H10A	108.4
N1^i^—Ni1—S1	163.99 (3)	S2—C10—H10A	108.4
C9—S1—Ni1	93.62 (4)	C11—C10—H10B	108.4
C9—S2—C10	103.11 (7)	S2—C10—H10B	108.4
C7—N1—N2	114.09 (10)	H10A—C10—H10B	107.5
C7—N1—Ni1	128.55 (9)	C10—C11—C12	111.85 (12)
N2—N1—Ni1	117.34 (8)	C10—C11—H11A	109.2
C9—N2—N1	110.70 (10)	C12—C11—H11A	109.2
C2—C1—C6	120.22 (13)	C10—C11—H11B	109.2
C2—C1—H1	119.9	C12—C11—H11B	109.2
C6—C1—H1	119.9	H11A—C11—H11B	107.9
C3—C2—C1	120.23 (13)	C13—C12—C11	113.67 (12)
C3—C2—H2	119.9	C13—C12—H12A	108.8
C1—C2—H2	119.9	C11—C12—H12A	108.8
C2—C3—C4	120.03 (13)	C13—C12—H12B	108.8
C2—C3—H3	120.0	C11—C12—H12B	108.8
C4—C3—H3	120.0	H12A—C12—H12B	107.7
C3—C4—C5	120.20 (13)	C14—C13—C12	113.20 (13)
C3—C4—H4	119.9	C14—C13—H13A	108.9
C5—C4—H4	119.9	C12—C13—H13A	108.9
C4—C5—C6	120.18 (13)	C14—C13—H13B	108.9
C4—C5—H5	119.9	C12—C13—H13B	108.9
C6—C5—H5	119.9	H13A—C13—H13B	107.8
C5—C6—C1	119.09 (12)	C13—C14—C15	113.56 (14)
C5—C6—C7	120.78 (11)	C13—C14—H14A	108.9
C1—C6—C7	120.07 (11)	C15—C14—H14A	108.9
N1—C7—C6	118.82 (11)	C13—C14—H14B	108.9
N1—C7—C8	122.19 (11)	C15—C14—H14B	108.9
C6—C7—C8	118.96 (11)	H14A—C14—H14B	107.7
C7—C8—H8A	109.5	C14—C15—H15A	109.5
C7—C8—H8B	109.5	C14—C15—H15B	109.5
H8A—C8—H8B	109.5	H15A—C15—H15B	109.5
C7—C8—H8C	109.5	C14—C15—H15C	109.5
H8A—C8—H8C	109.5	H15A—C15—H15C	109.5
H8B—C8—H8C	109.5	H15B—C15—H15C	109.5

Symmetry code: (i) −*x*+1, *y*, −*z*+3/2.

### Bis[S-n-hexyl 3-(1-phenylethylidene)dithiocarbazato-κ2N3,S]nickel(II) (I) Hydrogen-bond geometry (Å, º)

*Cg* is the centroid of the C1–C6 ring.

**Table:**

*D*—H···*A*	*D*—H	H···*A*	*D*···*A*	*D*—H···*A*
C14—H14*A*···*Cg*^ii^	0.99	2.75	3.5892 (18)	143

Symmetry code: (ii) −*x*+1, −*y*+1, −*z*+1.

### Bis[S-n-hexyl 3-(1-phenylethylidene)dithiocarbazato-κ2N3,S]copper(II) (II) Crystal data

**Table:**

[Cu(C_15_H_21_N_2_S_2_)_2_]	*F*(000) = 1372
*M**_r_* = 650.45	*D*_x_ = 1.334 Mg m^−^^3^
Monoclinic, *C**c*	Mo *K*α radiation, λ = 0.71075 Å
*a* = 22.7441 (7) Å	Cell parameters from 4858 reflections
*b* = 8.8636 (3) Å	θ = 3.3–27.4°
*c* = 17.0117 (6) Å	µ = 0.96 mm^−^^1^
β = 109.158 (1)°	*T* = 173 K
*V* = 3239.53 (19) Å^3^	Platelet, brown
*Z* = 4	0.23 × 0.10 × 0.03 mm

### Bis[S-n-hexyl 3-(1-phenylethylidene)dithiocarbazato-κ2N3,S]copper(II) (II) Data collection

**Table:**

Rigaku R-AXIS RAPID diffractometer	6505 reflections with *I* > 2σ(*I*)
Detector resolution: 10.000 pixels mm^-1^	*R*_int_ = 0.025
ω scans	θ_max_ = 27.5°, θ_min_ = 3.3°
Absorption correction: multi-scan (ABSCOR; Rigaku, 1995)	*h* = −29→29
*T*_min_ = 0.772, *T*_max_ = 0.976	*k* = −11→11
7274 measured reflections	*l* = −22→22
7274 independent reflections	

### Bis[S-n-hexyl 3-(1-phenylethylidene)dithiocarbazato-κ2N3,S]copper(II) (II) Refinement

**Table:**

Refinement on *F*^2^	Secondary atom site location: difference Fourier map
Least-squares matrix: full	Hydrogen site location: inferred from neighbouring sites
*R*[*F*^2^ > 2σ(*F*^2^)] = 0.031	H-atom parameters constrained
*wR*(*F*^2^) = 0.074	*w* = 1/[σ^2^(*F*_o_^2^) + (0.0443*P*)^2^] where *P* = (*F*_o_^2^ + 2*F*_c_^2^)/3
*S* = 1.03	(Δ/σ)_max_ < 0.001
7274 reflections	Δρ_max_ = 0.70 e Å^−^^3^
357 parameters	Δρ_min_ = −0.22 e Å^−^^3^
2 restraints	Absolute structure: Refined as an inversion twin
Primary atom site location: structure-invariant direct methods	Absolute structure parameter: 0.482 (10)

### Bis[S-n-hexyl 3-(1-phenylethylidene)dithiocarbazato-κ2N3,S]copper(II) (II) Special details

**Table:**

Geometry. All esds (except the esd in the dihedral angle between two l.s. planes) are estimated using the full covariance matrix. The cell esds are taken into account individually in the estimation of esds in distances, angles and torsion angles; correlations between esds in cell parameters are only used when they are defined by crystal symmetry. An approximate (isotropic) treatment of cell esds is used for estimating esds involving l.s. planes.
Refinement. Refined as a two-component inversion twin

### Bis[S-n-hexyl 3-(1-phenylethylidene)dithiocarbazato-κ2N3,S]copper(II) (II) Fractional atomic coordinates and isotropic or equivalent isotropic displacement parameters (Å^2^)

**Table:**

	*x*	*y*	*z*	*U*_iso_*/*U*_eq_	
Cu1	0.76372 (2)	0.47483 (4)	0.64581 (2)	0.02901 (10)	
S1	0.72319 (5)	0.66910 (10)	0.56233 (6)	0.0427 (2)	
S2	0.65326 (5)	0.65000 (14)	0.38556 (6)	0.0541 (3)	
S3	0.80973 (4)	0.60386 (9)	0.76328 (5)	0.0377 (2)	
S4	0.87369 (5)	0.46750 (11)	0.92560 (6)	0.0422 (2)	
N1	0.76458 (13)	0.3684 (3)	0.54100 (16)	0.0298 (6)	
N2	0.72530 (14)	0.4255 (4)	0.46522 (18)	0.0372 (7)	
N3	0.75419 (12)	0.3083 (3)	0.72177 (16)	0.0283 (6)	
N4	0.79439 (13)	0.3123 (3)	0.80456 (16)	0.0320 (6)	
C1	0.83534 (16)	0.1791 (4)	0.6128 (2)	0.0328 (7)	
C2	0.83552 (19)	0.0237 (4)	0.6288 (3)	0.0449 (9)	
H2	0.8092	−0.0422	0.5884	0.054*	
C3	0.8741 (2)	−0.0326 (5)	0.7033 (3)	0.0593 (13)	
H3	0.8732	−0.1374	0.7144	0.071*	
C4	0.9139 (2)	0.0595 (6)	0.7618 (3)	0.0577 (12)	
H4	0.9401	0.0185	0.8129	0.069*	
C5	0.91577 (18)	0.2123 (5)	0.7460 (3)	0.0498 (9)	
H5	0.9436	0.2763	0.7860	0.060*	
C6	0.87702 (16)	0.2717 (4)	0.6720 (2)	0.0366 (8)	
H6	0.8787	0.3764	0.6611	0.044*	
C7	0.79159 (15)	0.2419 (4)	0.5351 (2)	0.0339 (7)	
C8	0.7800 (2)	0.1604 (5)	0.4547 (2)	0.0489 (10)	
H8A	0.7354	0.1395	0.4299	0.073*	
H8B	0.8031	0.0651	0.4648	0.073*	
H8C	0.7938	0.2230	0.4166	0.073*	
C9	0.70540 (17)	0.5602 (5)	0.4723 (2)	0.0389 (8)	
C10	0.6477 (2)	0.5274 (5)	0.2988 (3)	0.0504 (10)	
H10A	0.6293	0.5855	0.2468	0.061*	
H10B	0.6904	0.4983	0.3016	0.061*	
C11	0.6101 (2)	0.3855 (6)	0.2931 (3)	0.0636 (13)	
H11A	0.5700	0.4110	0.3010	0.076*	
H11B	0.6329	0.3158	0.3383	0.076*	
C12	0.5971 (2)	0.3049 (5)	0.2076 (3)	0.0570 (11)	
H12A	0.6346	0.3124	0.1906	0.068*	
H12B	0.5892	0.1965	0.2143	0.068*	
C13	0.54203 (19)	0.3702 (5)	0.1394 (2)	0.0470 (9)	
H13A	0.5509	0.4772	0.1308	0.056*	
H13B	0.5051	0.3677	0.1578	0.056*	
C14	0.5267 (2)	0.2878 (5)	0.0568 (3)	0.0598 (11)	
H14A	0.5221	0.1788	0.0661	0.072*	
H14B	0.5618	0.2997	0.0350	0.072*	
C15	0.4673 (2)	0.3451 (5)	−0.0081 (3)	0.0626 (12)	
H15A	0.4326	0.3366	0.0137	0.094*	
H15B	0.4584	0.2845	−0.0589	0.094*	
H15C	0.4728	0.4509	−0.0209	0.094*	
C16	0.67196 (15)	0.1824 (4)	0.6188 (2)	0.0299 (7)	
C17	0.65939 (18)	0.0441 (4)	0.5760 (2)	0.0390 (8)	
H17	0.6801	−0.0451	0.6016	0.047*	
C18	0.6168 (2)	0.0384 (4)	0.4966 (3)	0.0469 (10)	
H18	0.6090	−0.0543	0.4670	0.056*	
C19	0.58533 (18)	0.1674 (5)	0.4601 (2)	0.0453 (9)	
H19	0.5562	0.1628	0.4053	0.054*	
C20	0.59591 (16)	0.3024 (4)	0.5024 (2)	0.0395 (8)	
H20	0.5736	0.3902	0.4773	0.047*	
C21	0.63895 (15)	0.3099 (4)	0.5813 (2)	0.0316 (7)	
H21	0.6461	0.4033	0.6104	0.038*	
C22	0.71886 (15)	0.1898 (4)	0.7031 (2)	0.0299 (7)	
C23	0.72371 (19)	0.0637 (4)	0.7638 (2)	0.0434 (9)	
H23A	0.7662	0.0234	0.7822	0.065*	
H23B	0.6944	−0.0165	0.7368	0.065*	
H23C	0.7137	0.1019	0.8120	0.065*	
C24	0.82018 (17)	0.4433 (4)	0.8252 (2)	0.0338 (8)	
C25	0.87310 (19)	0.2823 (4)	0.9736 (2)	0.0451 (9)	
H25A	0.8390	0.2782	0.9978	0.054*	
H25B	0.8659	0.2022	0.9309	0.054*	
C26	0.93557 (19)	0.2566 (5)	1.0418 (2)	0.0471 (9)	
H26A	0.9364	0.1523	1.0630	0.057*	
H26B	0.9691	0.2650	1.0167	0.057*	
C27	0.94983 (18)	0.3651 (5)	1.1151 (2)	0.0408 (8)	
H27A	0.9467	0.4700	1.0941	0.049*	
H27B	0.9183	0.3519	1.1432	0.049*	
C28	1.01432 (18)	0.3406 (5)	1.1781 (2)	0.0501 (10)	
H28A	1.0200	0.2315	1.1909	0.060*	
H28B	1.0458	0.3709	1.1524	0.060*	
C29	1.02664 (18)	0.4269 (5)	1.2592 (2)	0.0477 (9)	
H29A	1.0719	0.4248	1.2901	0.057*	
H29B	1.0143	0.5335	1.2462	0.057*	
C30	0.99290 (19)	0.3655 (5)	1.3133 (2)	0.0534 (10)	
H30A	0.9480	0.3716	1.2842	0.080*	
H30B	1.0036	0.4245	1.3648	0.080*	
H30C	1.0048	0.2600	1.3266	0.080*	

### Bis[S-n-hexyl 3-(1-phenylethylidene)dithiocarbazato-κ2N3,S]copper(II) (II) Atomic displacement parameters (Å^2^)

**Table:**

	*U*^11^	*U*^22^	*U*^33^	*U*^12^	*U*^13^	*U*^23^
Cu1	0.03446 (19)	0.02179 (16)	0.03268 (19)	−0.00067 (18)	0.01359 (15)	−0.00063 (18)
S1	0.0537 (6)	0.0254 (4)	0.0478 (5)	0.0061 (4)	0.0151 (4)	0.0052 (4)
S2	0.0525 (6)	0.0622 (7)	0.0448 (5)	0.0179 (5)	0.0124 (4)	0.0183 (5)
S3	0.0465 (5)	0.0260 (4)	0.0391 (5)	−0.0078 (4)	0.0120 (4)	−0.0041 (3)
S4	0.0466 (5)	0.0408 (5)	0.0335 (5)	−0.0107 (4)	0.0054 (4)	−0.0032 (4)
N1	0.0329 (13)	0.0283 (13)	0.0301 (13)	0.0021 (11)	0.0129 (11)	0.0022 (11)
N2	0.0380 (16)	0.0435 (17)	0.0314 (14)	0.0007 (14)	0.0129 (12)	0.0036 (13)
N3	0.0305 (14)	0.0255 (13)	0.0300 (13)	−0.0037 (11)	0.0115 (11)	−0.0044 (11)
N4	0.0349 (14)	0.0304 (14)	0.0312 (14)	−0.0054 (12)	0.0115 (11)	−0.0032 (11)
C1	0.0354 (18)	0.0269 (16)	0.0435 (19)	0.0024 (14)	0.0227 (15)	0.0011 (15)
C2	0.051 (2)	0.0243 (16)	0.066 (3)	0.0055 (16)	0.027 (2)	0.0049 (18)
C3	0.064 (3)	0.039 (2)	0.085 (3)	0.012 (2)	0.039 (3)	0.020 (2)
C4	0.052 (2)	0.062 (3)	0.062 (3)	0.025 (2)	0.024 (2)	0.025 (2)
C5	0.038 (2)	0.060 (2)	0.051 (2)	0.0027 (19)	0.0147 (17)	0.001 (2)
C6	0.0342 (17)	0.0313 (16)	0.049 (2)	0.0042 (15)	0.0195 (15)	0.0038 (16)
C7	0.0374 (17)	0.0298 (16)	0.0390 (18)	−0.0010 (15)	0.0189 (14)	−0.0029 (14)
C8	0.059 (2)	0.045 (2)	0.044 (2)	0.0051 (19)	0.0178 (19)	−0.0100 (18)
C9	0.0375 (19)	0.0437 (19)	0.0378 (19)	0.0053 (17)	0.0155 (16)	0.0115 (17)
C10	0.041 (2)	0.067 (3)	0.043 (2)	0.0022 (19)	0.0135 (17)	0.0181 (19)
C11	0.053 (2)	0.082 (3)	0.053 (3)	−0.010 (2)	0.014 (2)	0.027 (2)
C12	0.056 (3)	0.049 (2)	0.067 (3)	−0.002 (2)	0.022 (2)	0.013 (2)
C13	0.046 (2)	0.049 (2)	0.053 (2)	−0.0027 (19)	0.0261 (18)	0.0005 (19)
C14	0.069 (3)	0.047 (2)	0.072 (3)	−0.002 (2)	0.034 (2)	−0.008 (2)
C15	0.078 (3)	0.055 (3)	0.055 (3)	−0.009 (2)	0.024 (2)	−0.014 (2)
C16	0.0310 (16)	0.0278 (15)	0.0340 (16)	−0.0067 (13)	0.0149 (13)	−0.0032 (13)
C17	0.045 (2)	0.0290 (18)	0.044 (2)	−0.0014 (16)	0.0158 (16)	−0.0034 (16)
C18	0.052 (2)	0.041 (2)	0.046 (2)	−0.0160 (19)	0.0134 (18)	−0.0131 (18)
C19	0.043 (2)	0.053 (2)	0.0363 (19)	−0.0130 (18)	0.0079 (16)	−0.0038 (17)
C20	0.0335 (17)	0.043 (2)	0.0417 (19)	−0.0036 (16)	0.0125 (15)	0.0017 (17)
C21	0.0328 (16)	0.0302 (16)	0.0348 (17)	−0.0050 (14)	0.0150 (13)	−0.0046 (14)
C22	0.0339 (17)	0.0264 (15)	0.0333 (16)	−0.0009 (13)	0.0163 (13)	−0.0024 (13)
C23	0.051 (2)	0.0363 (18)	0.042 (2)	−0.0107 (18)	0.0138 (17)	0.0035 (17)
C24	0.0337 (18)	0.0371 (19)	0.0301 (17)	−0.0023 (15)	0.0098 (14)	−0.0044 (15)
C25	0.055 (2)	0.0394 (19)	0.0387 (19)	−0.0092 (18)	0.0131 (17)	−0.0028 (16)
C26	0.054 (2)	0.048 (2)	0.0388 (19)	0.0107 (19)	0.0141 (17)	−0.0030 (18)
C27	0.044 (2)	0.041 (2)	0.0378 (18)	0.0061 (17)	0.0138 (16)	−0.0044 (16)
C28	0.042 (2)	0.061 (3)	0.044 (2)	0.0095 (19)	0.0111 (17)	−0.0032 (19)
C29	0.039 (2)	0.050 (2)	0.046 (2)	−0.0037 (18)	0.0040 (16)	−0.0035 (19)
C30	0.047 (2)	0.065 (3)	0.045 (2)	−0.010 (2)	0.0096 (17)	−0.008 (2)

### Bis[S-n-hexyl 3-(1-phenylethylidene)dithiocarbazato-κ2N3,S]copper(II) (II) Geometric parameters (Å, º)

**Table:**

Cu1—N1	2.023 (3)	C13—H13A	0.9900
Cu1—N3	2.020 (3)	C13—H13B	0.9900
Cu1—S1	2.2299 (9)	C14—C15	1.524 (7)
Cu1—S3	2.2414 (9)	C14—H14A	0.9900
S1—C9	1.742 (4)	C14—H14B	0.9900
S2—C9	1.752 (4)	C15—H15A	0.9800
S2—C10	1.804 (5)	C15—H15B	0.9800
S3—C24	1.740 (4)	C15—H15C	0.9800
S4—C24	1.755 (4)	C16—C21	1.391 (5)
S4—C25	1.835 (4)	C16—C17	1.406 (5)
N1—C7	1.298 (4)	C16—C22	1.481 (4)
N1—N2	1.400 (4)	C17—C18	1.381 (5)
N2—C9	1.296 (5)	C17—H17	0.9500
N3—C22	1.297 (4)	C18—C19	1.383 (6)
N3—N4	1.406 (4)	C18—H18	0.9500
N4—C24	1.296 (5)	C19—C20	1.376 (5)
C1—C6	1.399 (5)	C19—H19	0.9500
C1—C2	1.404 (5)	C20—C21	1.380 (5)
C1—C7	1.478 (5)	C20—H20	0.9500
C2—C3	1.377 (7)	C21—H21	0.9500
C2—H2	0.9500	C22—C23	1.501 (5)
C3—C4	1.373 (7)	C23—H23A	0.9800
C3—H3	0.9500	C23—H23B	0.9800
C4—C5	1.384 (7)	C23—H23C	0.9800
C4—H4	0.9500	C25—C26	1.528 (5)
C5—C6	1.383 (5)	C25—H25A	0.9900
C5—H5	0.9500	C25—H25B	0.9900
C6—H6	0.9500	C26—C27	1.523 (5)
C7—C8	1.491 (5)	C26—H26A	0.9900
C8—H8A	0.9800	C26—H26B	0.9900
C8—H8B	0.9800	C27—C28	1.522 (5)
C8—H8C	0.9800	C27—H27A	0.9900
C10—C11	1.506 (6)	C27—H27B	0.9900
C10—H10A	0.9900	C28—C29	1.521 (6)
C10—H10B	0.9900	C28—H28A	0.9900
C11—C12	1.559 (7)	C28—H28B	0.9900
C11—H11A	0.9900	C29—C30	1.481 (6)
C11—H11B	0.9900	C29—H29A	0.9900
C12—C13	1.515 (6)	C29—H29B	0.9900
C12—H12A	0.9900	C30—H30A	0.9800
C12—H12B	0.9900	C30—H30B	0.9800
C13—C14	1.519 (6)	C30—H30C	0.9800
			
S1—Cu1—S3	98.53 (4)	C13—C14—H14B	109.0
N1—Cu1—S1	85.43 (8)	C15—C14—H14B	109.0
N3—Cu1—S1	149.66 (8)	H14A—C14—H14B	107.8
N1—Cu1—S3	152.51 (8)	C14—C15—H15A	109.5
N3—Cu1—S3	85.37 (8)	C14—C15—H15B	109.5
N1—Cu1—N3	104.90 (11)	H15A—C15—H15B	109.5
C9—S1—Cu1	93.45 (13)	C14—C15—H15C	109.5
C9—S2—C10	105.16 (19)	H15A—C15—H15C	109.5
C24—S3—Cu1	93.07 (12)	H15B—C15—H15C	109.5
C24—S4—C25	102.70 (18)	C21—C16—C17	118.9 (3)
C7—N1—N2	114.6 (3)	C21—C16—C22	121.1 (3)
C7—N1—Cu1	127.8 (2)	C17—C16—C22	120.0 (3)
N2—N1—Cu1	117.1 (2)	C18—C17—C16	119.8 (3)
C9—N2—N1	112.6 (3)	C18—C17—H17	120.1
C22—N3—N4	114.4 (3)	C16—C17—H17	120.1
C22—N3—Cu1	128.5 (2)	C17—C18—C19	120.2 (3)
N4—N3—Cu1	116.84 (18)	C17—C18—H18	119.9
C24—N4—N3	112.5 (3)	C19—C18—H18	119.9
C6—C1—C2	118.6 (3)	C20—C19—C18	120.5 (3)
C6—C1—C7	121.4 (3)	C20—C19—H19	119.7
C2—C1—C7	120.0 (3)	C18—C19—H19	119.7
C3—C2—C1	119.6 (4)	C19—C20—C21	119.9 (3)
C3—C2—H2	120.2	C19—C20—H20	120.1
C1—C2—H2	120.2	C21—C20—H20	120.1
C4—C3—C2	121.4 (4)	C20—C21—C16	120.7 (3)
C4—C3—H3	119.3	C20—C21—H21	119.7
C2—C3—H3	119.3	C16—C21—H21	119.7
C3—C4—C5	119.8 (4)	N3—C22—C16	117.7 (3)
C3—C4—H4	120.1	N3—C22—C23	122.3 (3)
C5—C4—H4	120.1	C16—C22—C23	120.0 (3)
C6—C5—C4	119.9 (4)	C22—C23—H23A	109.5
C6—C5—H5	120.1	C22—C23—H23B	109.5
C4—C5—H5	120.1	H23A—C23—H23B	109.5
C5—C6—C1	120.7 (3)	C22—C23—H23C	109.5
C5—C6—H6	119.6	H23A—C23—H23C	109.5
C1—C6—H6	119.6	H23B—C23—H23C	109.5
N1—C7—C1	116.9 (3)	N4—C24—S3	127.5 (3)
N1—C7—C8	122.9 (3)	N4—C24—S4	118.7 (3)
C1—C7—C8	120.2 (3)	S3—C24—S4	113.7 (2)
C7—C8—H8A	109.5	C26—C25—S4	109.3 (3)
C7—C8—H8B	109.5	C26—C25—H25A	109.8
H8A—C8—H8B	109.5	S4—C25—H25A	109.8
C7—C8—H8C	109.5	C26—C25—H25B	109.8
H8A—C8—H8C	109.5	S4—C25—H25B	109.8
H8B—C8—H8C	109.5	H25A—C25—H25B	108.3
N2—C9—S1	127.2 (3)	C27—C26—C25	115.0 (3)
N2—C9—S2	120.1 (3)	C27—C26—H26A	108.5
S1—C9—S2	112.7 (2)	C25—C26—H26A	108.5
C11—C10—S2	116.2 (3)	C27—C26—H26B	108.5
C11—C10—H10A	108.2	C25—C26—H26B	108.5
S2—C10—H10A	108.2	H26A—C26—H26B	107.5
C11—C10—H10B	108.2	C28—C27—C26	112.6 (3)
S2—C10—H10B	108.2	C28—C27—H27A	109.1
H10A—C10—H10B	107.4	C26—C27—H27A	109.1
C10—C11—C12	111.9 (4)	C28—C27—H27B	109.1
C10—C11—H11A	109.2	C26—C27—H27B	109.1
C12—C11—H11A	109.2	H27A—C27—H27B	107.8
C10—C11—H11B	109.2	C29—C28—C27	114.5 (3)
C12—C11—H11B	109.2	C29—C28—H28A	108.6
H11A—C11—H11B	107.9	C27—C28—H28A	108.6
C13—C12—C11	113.3 (4)	C29—C28—H28B	108.6
C13—C12—H12A	108.9	C27—C28—H28B	108.6
C11—C12—H12A	108.9	H28A—C28—H28B	107.6
C13—C12—H12B	108.9	C30—C29—C28	113.6 (4)
C11—C12—H12B	108.9	C30—C29—H29A	108.8
H12A—C12—H12B	107.7	C28—C29—H29A	108.8
C12—C13—C14	113.9 (4)	C30—C29—H29B	108.8
C12—C13—H13A	108.8	C28—C29—H29B	108.8
C14—C13—H13A	108.8	H29A—C29—H29B	107.7
C12—C13—H13B	108.8	C29—C30—H30A	109.5
C14—C13—H13B	108.8	C29—C30—H30B	109.5
H13A—C13—H13B	107.7	H30A—C30—H30B	109.5
C13—C14—C15	112.9 (4)	C29—C30—H30C	109.5
C13—C14—H14A	109.0	H30A—C30—H30C	109.5
C15—C14—H14A	109.0	H30B—C30—H30C	109.5
